# Supplementary material for: Decreased cardiac pacemaking and attenuated β-adrenergic response in TRIC-A knockout mice
Source: PLoS One. 2020 Dec 21;15(12):e0244254. doi: 10.1371/journal.pone.0244254 (PMC7751866; doi:10.1371/journal.pone.0244254)
Supplement: S1 File — (PDF) [file pone.0244254.s001.pdf]

	For	Rev
β actin	CAACTGGGACGACATGGAGAA	CAGCCTGGATGGCTACGTACA
TRIC-A	CCACCAAGGCCAGTCTGTAT	AGCACTGGGCAGATGTAACC
TRIC-B	TGAAGATGTCCTTCCCTTGC	AGGGTGTCCTCAAAGGGAGT
β 1	CATCGTTCTGCTCATCGTGG	ACACACAGCACATCTACCGA
β 2	GAGCGACTACAAACCGTCAC	TGGTACTTGAAGGGCGATGT
HCN2	TCCAGTCCCTGGATTCGTCA	AGTTCACAATCTCCTCACGCA
HCN4	GGACCGCATAGGCAAGAAGA	GGCCACCGAAGTAGTAGCAG
SERCA1	ACCAGATGTCAGTTTGCA	CAAGGTGGTGAGAGCAG
SERCA2	TGGCAGCATGTATATCTTTC	CTTTAATTCGTTGCACACTC
SERCA3	AATGTTGGCGAGGTTGTC	CAATAGCCAAATAGCGGAA
RyR1	AAGACGAAGCTGCCTAAGA	GGCTGTCCCTATTGCTG
RyR2	ATGTCGCTTGAAACCCTC	GATGCCGTAAGTCCAGC
RyR3	ACTGCCCTCCCTAAACAAAG	CACAGCCGAACATAAATACCTA
CaV1.2	CCCTTCTTGTGCTCTTCGTCATCA	GGCTTTGGCTTTCTCCCTCTCT
Cav1.3	ACTACAACTTTCCGCTCGGT	CTTGCATAGTTTGCCTCTGC
NCX1	CCTTGTGCATCTTAGCAATG	TCTCACTCATCTCCACCAGA
M2	GGTAAGGACTGTGGAAGA	ACCAGGCATGTTGTTGTTG
IP3R2	AATGTTGGCGAGGTTGTC	CAATAGCCAAATAGCGGAA
STIM1	TGGGATCTCAGAGGGATTTG	GGGGCTAAGAGAATGGGAAG
Orai1	TTACTCCGAGGTGATGAGCC	TGGTGGGTAGTCATGGTCTG
Orai2	AACCTCAACTCCATCAGCGA	GACCACGAAGATGAGACCCA
PLN	TACCTCACTCGCTCGGCTAT	GATGCAGATCAGCAGCAGAC

Supporting Information Table 1

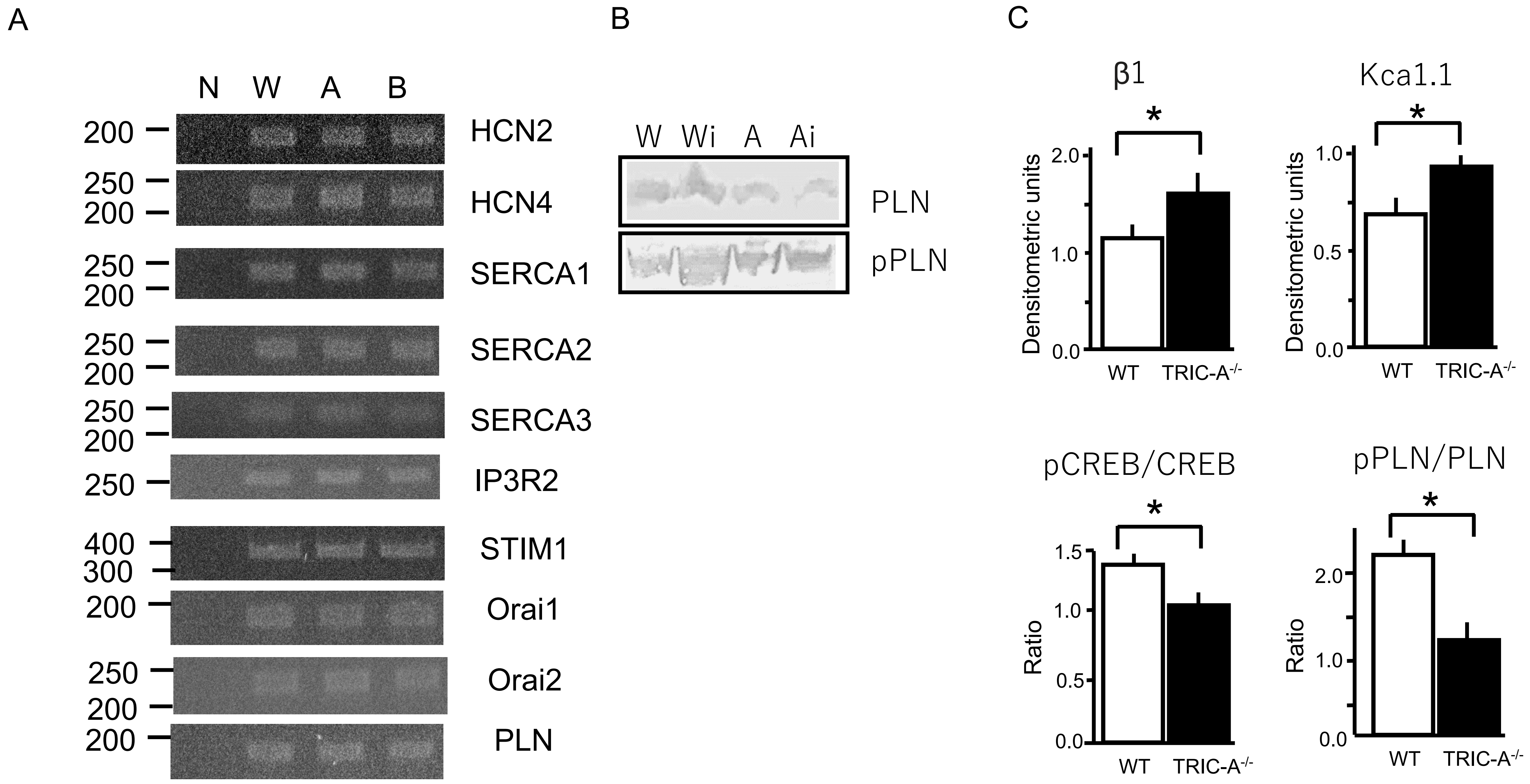

Supporting Information Figure 1

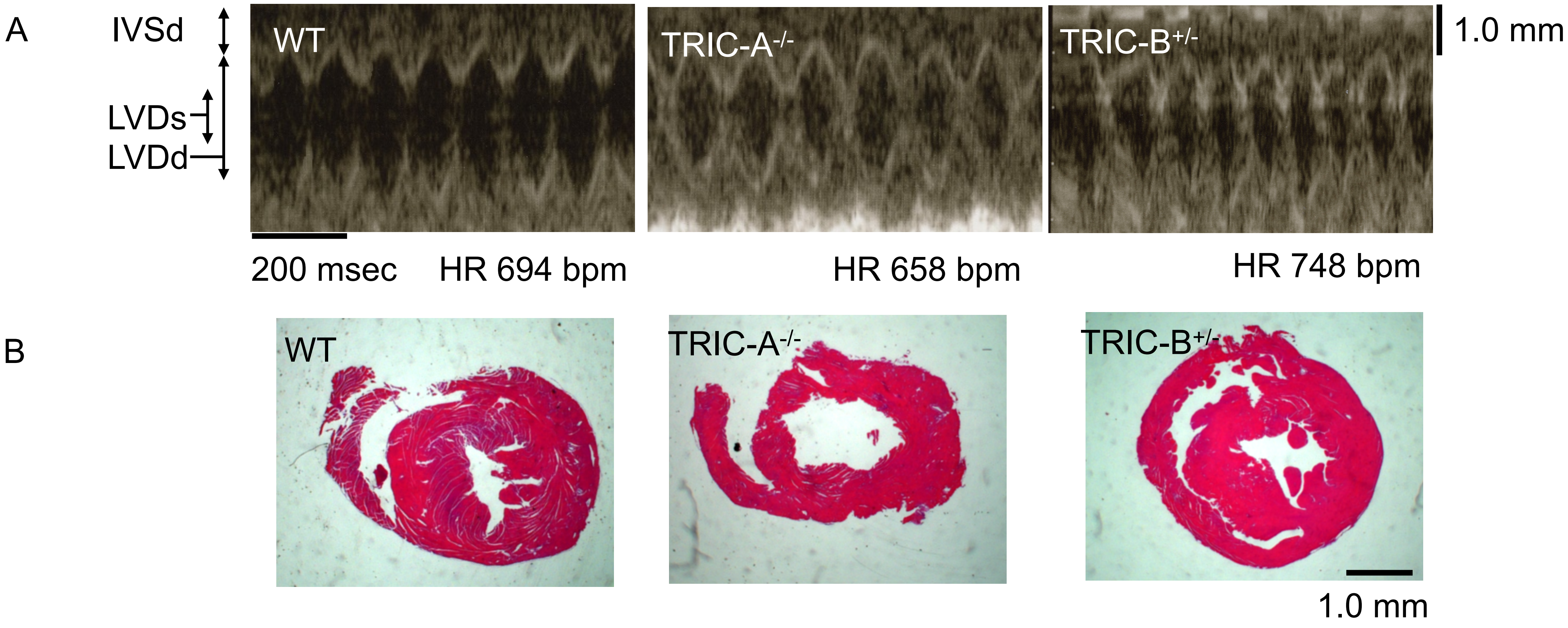

Supporting Information Figure 2

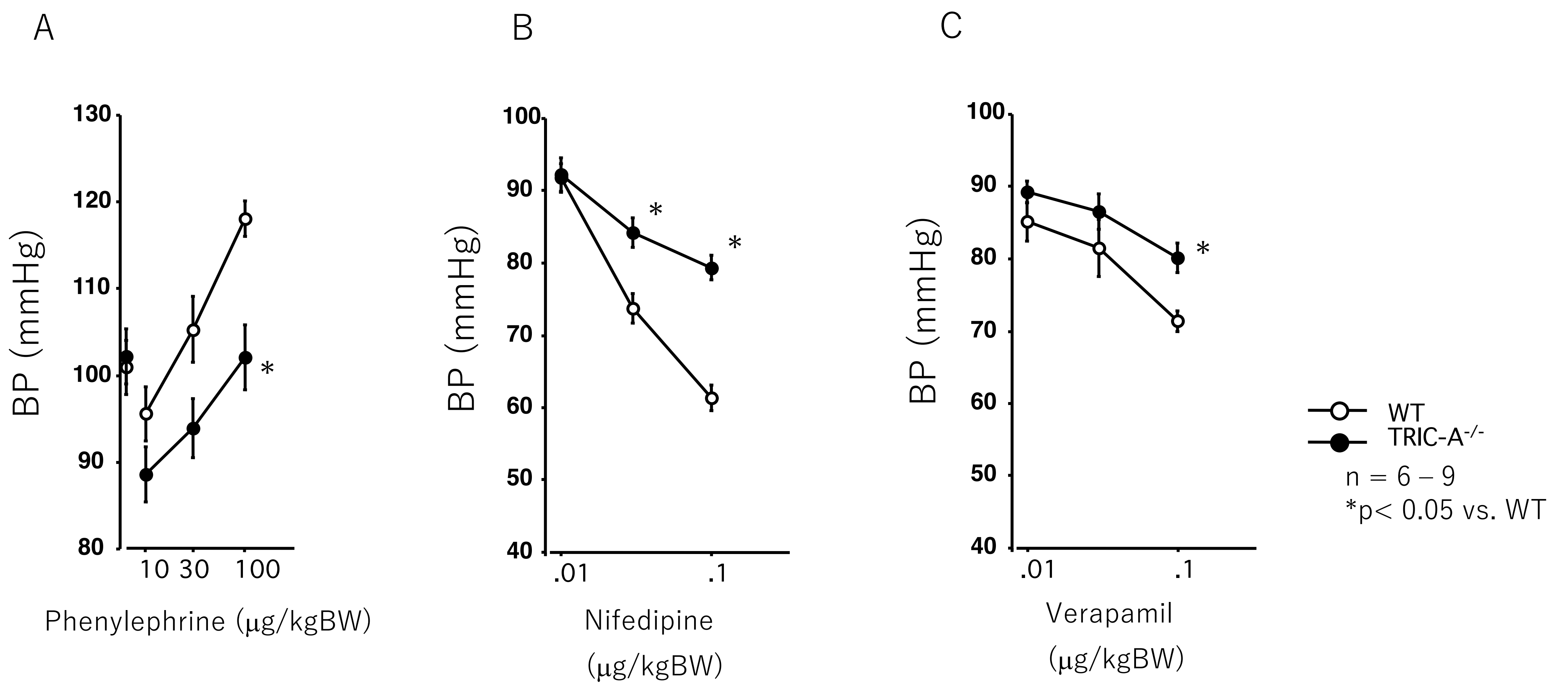

Supporting Information Figure 3

A TRIC-A$^{-/-}$ sinus pause (electrocardiography)

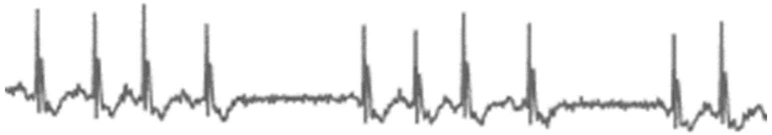

B TRIC-A$^{-/-}$ atrioventricular block (electrocardiography)

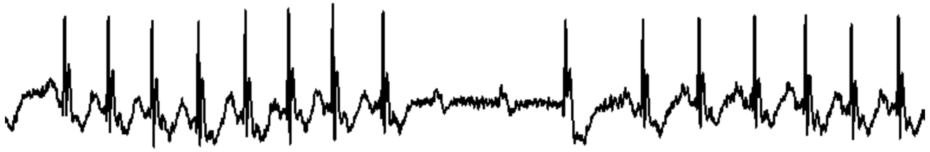

Supporting Information Figure 4

A Original RT-PCR Gels

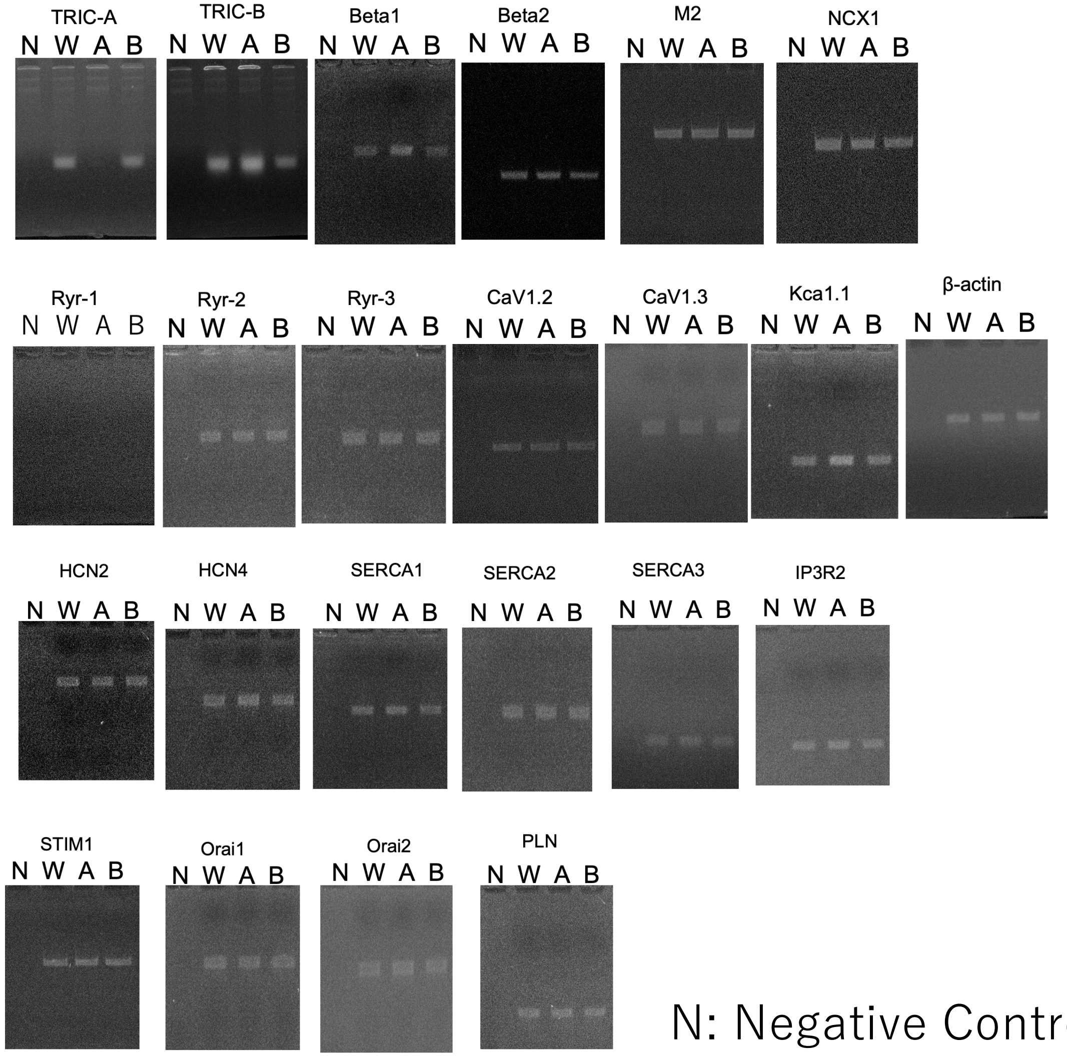

N: Negative Control
W: wild-type
A: TRIC-A$^{-/-}$
B: TRIC-B$^{+/-}$

B Original Western Blots

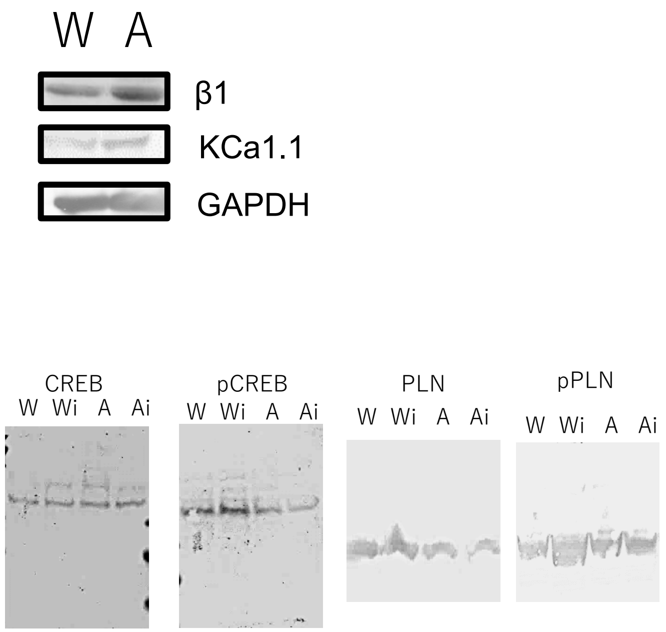

W: wild-type
A: TRIC-A$^{-/-}$
i: isoproterenol

Supporting Information Figure 5
