# Supplementary material for: Decreased cardiac pacemaking and attenuated β-adrenergic response in TRIC-A knockout mice
Source: PLoS One. 2020 Dec 21;15(12):e0244254. doi: 10.1371/journal.pone.0244254 (PMC7751866; doi:10.1371/journal.pone.0244254)
Supplement: S1 Text — (DOCX) [file pone.0244254.s002.docx]

**S1 Text.**

# Detailed Materials and Methods

## Model animals

The animals were housed under a constant 12 h light/dark cycle with free access to food and water throughout the study. Wild-type (WT), TRIC-A^–/–^, and TRIC-B^+/–^ mice, 12–20 weeks old, were used in all experiments. TRIC-A^–/–^ and TRIC-B^+/–^ mice were generated as described previously [4]. As reported previously, TRIC-B-null is lethal, so haplodeficient TRIC-B^+/–^ mice were used [4, 5].

## RNA isolation and RT-PCR

The sinus node was dissected under a dissection microscope with observation of cardiac automaticity (MZ8; Leica GmbH, Wetzler, Germany). Total RNA was extracted from the sinus nodes using an RNeasy Kit (Qiagen, Valencia, CA) [6]. Reverse transcription was performed with oligo-dT primer and SuperScript IV reverse transcriptase (Invitrogen, San Diego, CA). Comparative reverse transcription polymerase chain reaction (RT-PCR) was performed using EXTaq (Takara, Otsu, Japan) with 30–38 PCR cycles. Genes encoding TRIC-A, TRIC-B, β1- and β_2_-adrenergic receptors, muscarinic type-2 receptor (M2), sodium-calcium exchanger-1 (NCX1), ryanodine receptors (RyR1, RyR2, and RyR3), voltage-dependent calcium channels (CaV1.2 and CaV1.3), voltage-activated K^+^ (BK) channel (KCa1.1), and β-actin were amplified by PCR. Primers used in RT-PCR analyses are shown in Supporting Table 1. The results were normalized relative to the level of β-actin mRNA expression.

## Western blotting

Protein samples from the sinus nodes were homogenized and lysed in 50 mM Tris (pH 7.5), 140 mM NaCl, and 5 mM ethylenediaminetetraacetic acid (EDTA) with protease inhibitor cocktail (Complete Mini; Sigma-Aldrich, St. Louis, MO). The samples were resuspended in 2× sample buffer containing 4% SDS, 125 mM Tris-HCl (pH 6.8), 10% 2-mercaptoethanol, and 10% sucrose; 0.0004% Bromophenol blue was added, and the samples were incubated at 95°C for 3 min. The proteins were size-fractionated by sodium dodecyl sulfate (SDS)-polyacrylamide gel electrophoresis (PAGE) on 7.5% polyacrylamide gels. Then the proteins were transferred onto polyvinylidene difluoride (PVDF) membranes and blocked for 1 h at room temperature using 5% dried milk and Tris-buffered saline (TBS, pH 7.4) containing 0.1% Tween 20. The membranes were incubated with primary antibody to β1-adrenergic receptor (rabbit polyclonal antibody 1:200; Alomone Labs, Jerusalem, Israel), KCa1.1 (rabbit polyclonal antibody 1:500; Alomone Labs), cAMP-responsive element binding protein (CREB) (48H2) (rabbit monoclonal antibody 1:1000; Cell Signaling Technology, Santa Cruz, CA), phosphorylated CREB (pCREB) (Sr133) (87G3) (rabbit monoclonal antibody 1:1000, Cell Signaling Technology), phospholamban (PLN) (rabbit monoclonal antibody 1:1000, Cell Signaling Technology), phospho-phospholamban (PLN) (rabbit monoclonal antibody 1:400, Cell Signaling Technology), glyceraldehyde 3-phosphate dehydrogenase (GAPDH) (rabbit monoclonal antibody 1:10000; Abcam, Cambridge, UK), or goat anti-rabbit secondary antibody (1:7500; Promega Co., Ltd., Fitchburg, WI). Proteins were detected using a ProBlot system (Promega). To evaluate CREB and pCREB expression, the sinus nodes were excised 10 min after intraperitoneal injection of isoproterenol (0.5 mg/kg). The results were normalized relative to the level of GAPDH protein expression.

**Densitometric-Analysis of Western blots**

Western blotting analyses revealed an increase in the protein level of the β1-adrenergic receptor in TRIC-A^−/−^ sinus nodes compared with those of WT controls (1.08 ± 0.04 vs. 1.65 ± 0.10, *n* = 6; *P* < 0.05) (Fig. 1). The sinus nodes of TRIC-A^−/−^ mice also showed increased KCa1.1 protein expression compared with the sinus nodes of WT mice (0.92 ± 0.04 vs. 0.71 ± 0.05, *n* = 6; *P* < 0.05) (Fig. 1). GAPDH was used as an internal control. These results suggest the involvement of TRIC channels in sympathetic nerve regulation. Therefore, we examined increases in the pCREB level in the sinus node to evaluate the β-adrenergic response. Isoproterenol injection resulted in a significant increase in the pCREB level in WT SA nodes, whereas a marginal response was observed in the SA nodes of TRIC-A^−/−^ compared with WT mice (1.38 ± 0.08-fold vs. 1.03 ± 0.07-fold, *n* = 6; *P* < 0.05) (Fig. 1). Total CREB levels were also evaluated (CREB), and the results suggested a decreased β1-adrenergic response in TRIC-A^−/−^ SA nodes. We also examined phosphorylation of phospholamban (S1 Fig). Isoproterenol injection resulted in a significant increase in the phosphorylated phospholamban level in WT SA nodes, whereas a limited response was observed in the SA nodes of TRIC-A^−/−^ mice compared with WT controls (2.23 ± 0.06-fold vs. 1.17 ± 0.04-fold, *n* = 6; *P* < 0.05).

## Blood pressure measurement

All physiological studies were conducted between 10:00 and 16:00 at room temperature (23°C). The mice were kept in a warmed chamber at 37°C. Systolic blood pressure (SBP) was measured in conscious mice with the tail-cuff method (MK-1030; Muromachi Kikai Co., Ltd., Tokyo, Japan).

## Echocardiography

A commercial echocardiography machine equipped with a 10 MHz transducer (Aspen; Acuson, Stockton, CA) was used to record the M-mode echocardiogram in the mid-portion of the left ventricle. Three to five beats were averaged for each measurement. The heart was first imaged in two-dimensional (2D) mode in the parasternal short-axis view. From this view, an M-mode cursor was positioned perpendicular to the interventricular septum and posterior wall of the left ventricle (LV) at the level of the chordae tendineae. Echocardiographic recordings were analyzed to determine the LV inner diameter at end-diastole and systole and the LV ejection fraction using the software supplied with the system

## Electrocardiography

The mice were anesthetized as described previously [6] during implantation of the electrical lead into the back. Electrocardiography (ECG) was recorded with a preamplifier (MEG-5200; Nihon Kohden, Tokyo, Japan) through the electrical lead, digitized with a Power Lab system, and analyzed with LabChart 8 (AD Instruments, Dunedin, New Zealand). The HRV power spectrum consists of three components: very low frequency, low frequency (LF), and high frequency (HF). Generally, the LF component reflects sympathetic/parasympathetic tone, whereas the HF component reflects parasympathetic tone. The following ranges were specified for the spectral components according to the manufacturer’s protocol: very low frequency, < 0.15; LF, < 1.5; and HF, < 5.

## Inotropic atrial contraction

The right atria were dissected free of the ventricular tissue and placed in an oxygenated tissue bath filled with a Tyrode’s solution (123.8 mM NaCl, 5.0 mM KCl, 2.0 mM CaCl_2_, 1.2 mM MgCl_2_, 25.0 mM NaHCO_3_, and 11.2 mM glucose) at 23°C. After incubation for 10 min to stabilize basal contraction, the isometric contractile force was measured using a force transducer (CD200; Nihon Kohden), as described previously [7]. All data were acquired using a Power Lab system and analyzed with LabChart 8.

## Cardiac action potentials in the sinus node

The spontaneously beating right atrium was mounted in the organ bath (10 mL) and perfused continuously with oxygenated Tyrode’s solution at 23°C. For cardiac action potential recordings in the sinus node, the hearts were excised and rinsed in normal Tyrode’s solution. The sinus node region was dissected from the right atrium along the superior vena cava and crista terminalis (Fig. S5). After dissection, the spontaneously beating sinus node region was mounted in the organ bath. Intracellular action potentials were recorded from the intracardiac surface using conventional glass pipettes filled with 3.0 M KCl (tip resistance: 20–30 MΩ). Action potentials were recorded with an amplifier (MEZ-8301; Nihon Kohden) for analyses. All data were acquired using the Power Lab system and then analyzed with LabChart 8, as described previously [8].

# Results

## RT-PCR analyses in sinus node

In addition to RT-PCR analyses (Fig. 1A), we also examined HCN channels, which are thought to be involved in action potential formation, and sarco/endoplasmic reticulum Ca^2+^-ATPase (SERCA), which plays a significant role in calcium sequestration, and IP3-receptor type 2, which releases calcium from intracellular calcium stores, while no significant changes were observed.

**Western analysis of phospholamban**

As pCREB increase was not observed in TRIC-A^–/–^ SA node, we further examined increases in phosphor (p)-phospholamban in the sinus node to evaluate the β-adrenergic response. Isoproterenol injection resulted in a significant increase in phosphorylated-phospholamban level in the WT SA nodes, while a limited response was observed in TRIC-A^–/–^ SA nodes compared to WT (2.23 ± 0.06 vs. 1.17 ± 0.04-fold, *n* = 6, respectively; *P* < 0.05) (SIF 1B).

## Echocardiogram

As TRIC-A^–/–^ mice showed decreased HR, we analyzed echocardiograms. There were no differences among the three groups in the percent fractional shortening (%FS), suggesting conserved cardiac pump function. Further, the heart wall thickness was measured, but no significant changes were observed.

## Histological analyses

Hearts from 12-week-old WT, TRIC-A^–/–^, and TRIC-B^+/–^ mice were cut into transverse sections and stained with hematoxylin and eosin (HE). Gross examination of histological results with HE staining showed no apparent differences among the three groups. Overall, echocardiography and histological analyses showed no apparent hypotrophic effect in TRIC-A^–/–^ or TRIC-B^+/–^ mice.

## Effects of α1 agonist treatment (phenylephrine)

A previous study indicated that TRIC-A^–/–^ mice showed increased vascular resistance [5]. Therefore, we examined the effects of α1 agonist treatment as it would increase vascular tonus. If TRIC-A^–/–^ mice have a significant increase in vascular resistance, phenylephrine would be expected to show a limited effect on SBP. Phenylephrine increased SBP in a dose-dependent manner in WT mice. By contrast, TRIC-A^–/–^ mice showed decreased response to phenylephrine. These observations showed good agreement with the previous study, suggesting that TRIC-A^–/–^ mice have increased vascular resistance.

## Decreased responsiveness to calcium antagonists (nifedipine and verapamil)

To analyze the previously reported increased vascular resistance in TRIC-A^–/–^ mice, we examined the effects of calcium antagonists. Nifedipine (a dihydropyridine derivative) decreased SBP dose-dependently in WT mice, whereas TRIC-A^–/–^ mice exhibited limited responses. Verapamil, a phenylalkylamine derivative, also dose-dependently decreased the SBP in WT mice, whereas TRIC-A^–/–^ mice exhibited a decreased response. Overall, our blood pressure analyses revealed decreased responsiveness to α1 agonists and calcium antagonists. TRIC-A^–/–^ mice appear to have a modified SBP and HR regulatory system compared to WT mice.

# Figure Legends

**S1 Table.**

**Oligo DNAs used in RT-PCR analyses**

**S1 Fig.**

1. **RT-PCR**

RT-PCR analyses of the sinus nodes of WT (W), TRIC-A^–/–^ (A), and TRIC-B^+/–^ (B) mice (A). N, negative control. Primer sets are indicated.

1. **Western analysis of phospholamban (PLN) and phospho-phospholamban (p-PLN)**
2. **Western blot densitometry**

DensitomeTRIC-Analysis of the levels of the following proteins in the sinus nodes of WT and TRIC-A^−/−^ mice, as detected by Western blot analysis: β1-adrenergic receptor (β1), KCa1.1, phosphorylated CREB, total CREB, phosphorylated PLN, and total PLN (*n* = 6).

**S2 Fig.**

**Echocardiogram and histological examination**

**A.** Representative echocardiograms of the hearts (short-axis view) of WT, TRIC-A^–/–^, and TRIC-B^+/–^ mice. There were no significant differences in interventricular septum diameter (IVSd), LVDd, or LVDs among these mice.

**B.** HE staining of the hearts of WT, TRIC-A^–/–^, and TRIC-B^+/–^ mice. There were no significant differences in IVSd among these mice.

**S3 Fig.**

**SBP changes in response to phenylephrine, nifedipine, and verapamil**

Pharmacological responses to phenylephrine (A), nifedipine (B), and verapamil (C).

Dose-dependent SBP changes in WT mice and TRIC-A^–/–^ mice. Values are the mean ± standard error of the mean (*n* = 6–9). TRIC-A^–/–^ mice had a longer SDRR than WT mice. *P* < 0.05.

**S4 Fig.**

**ECG traces of arrhythmia in TRIC-A^–/–^ mice**

ECG traces of sinus pause (A) and AV block (B) in TRIC-A^–/–^ mice.

**S5 Fig.**

1. **Original RT-PCR gels**

Primer sets are indicated.

1. **Original Western blots**

Examined proteins are indicated.
