# Supplementary material for: Decreased cardiac pacemaking and attenuated β-adrenergic response in TRIC-A knockout mice
Source: PLoS One. 2020 Dec 21;15(12):e0244254. doi: 10.1371/journal.pone.0244254 (PMC7751866; doi:10.1371/journal.pone.0244254)
Supplement: Attachment — Submitted filename: ResponseToReviwers.docx [file pone.0244254.s003.docx]

*>>>>>>>*

*PONE-D-20-27381
Decreased cardiac pacemaking and attenuated β-adrenergic response in TRIC-A knockout mice
PLOS ONE

Dear Dr. Murakami,

Thank you for submitting your manuscript to PLOS ONE. After careful consideration, we feel that it has merit but does not fully meet PLOS ONE’s publication criteria as it currently stands. Therefore, we invite you to submit a revised version of the manuscript that addresses the points raised during the review process.

There are some technical issues that need to be addressed that are indicated below.

Please submit your revised manuscript by Dec 13 2020 11:59PM. If you will need more time than this to complete your revisions, please reply to this message or contact the journal office at plosone@plos.org. When you're ready to submit your revision, log on to https://www.editorialmanager.com/pone/ and select the 'Submissions Needing Revision' folder to locate your manuscript file.

Please include the following items when submitting your revised manuscript:*

- *A rebuttal letter that responds to each point raised by the academic editor and reviewer(s). You should upload this letter as a separate file labeled 'Response to Reviewers'.*
- *A marked-up copy of your manuscript that highlights changes made to the original version. You should upload this as a separate file labeled 'Revised Manuscript with Track Changes'.*
- *An unmarked version of your revised paper without tracked changes. You should upload this as a separate file labeled 'Manuscript'.*

*If you would like to make changes to your financial disclosure, please include your updated statement in your cover letter. Guidelines for resubmitting your figure files are available below the reviewer comments at the end of this letter.

If applicable, we recommend that you deposit your laboratory protocols in protocols.io to enhance the reproducibility of your results. Protocols.io assigns your protocol its own identifier (DOI) so that it can be cited independently in the future. For instructions see:* [*http://journals.plos.org/plosone/s/submission-guidelines#loc-laboratory-protocols*](http://journals.plos.org/plosone/s/submission-guidelines#loc-laboratory-protocols) *We look forward to receiving your revised manuscript.

Kind regards,

Agustín Guerrero-Hernandez
Academic Editor
PLOS ONE

Journal Requirements:*

*When submitting your revision, we need you to address these additional requirements.*

*1. Please ensure that your manuscript meets PLOS ONE's style requirements, including those for file naming. The PLOS ONE style templates can be found at*

*https://journals.plos.org/plosone/s/file?id=wjVg/PLOSOne_formatting_sample_main_body.pdf and*

*https://journals.plos.org/plosone/s/file?id=ba62/PLOSOne_formatting_sample_title_authors_affiliations.pdf*

*>>>>*

We have ensured that that the revised manuscript conforms to the style requirements of *PLoS ONE*.

*2. To comply with PLOS ONE submissions requirements, in your Methods section, please provide additional information on the animal research and ensure you have included details on (1) methods of sacrifice, (2) methods of anesthesia and/or analgesia, and (3) efforts to alleviate suffering.*

>>>>

Additional information has been provided regarding compliance with the regulations of the animal experiments.

*3. PLOS ONE now requires that authors provide the original uncropped and unadjusted images underlying all blot or gel results reported in a submission’s figures or Supporting Information files. This policy and the journal’s other requirements for blot/gel reporting and figure preparation are described in detail at https://journals.plos.org/plosone/s/figures#loc-blot-and-gel-reporting-requirements and https://journals.plos.org/plosone/s/figures#loc-preparing-figures-from-image-files. When you submit your revised manuscript, please ensure that your figures adhere fully to these guidelines and provide the original underlying images for all blot or gel data reported in your submission. See the following link for instructions on providing the original image data: https://journals.plos.org/plosone/s/figures#loc-original-images-for-blots-and-gels.*

*>>>>>>>*

As we did not have uncropped images of some RT-PCR gels, we repeated the RT-PCR experiments. The original uncropped and unadjusted images of the RT-PCR gels and Western blots are presented in our Supporting Information Figure 5F. The Western blots in Figure 1A (for β1, KCa1.1, and GAPDH) were cut from original transferred membranes and subjected to immunoreaction because of the relatively low dilution of antibodies (200–500-fold). Therefore, they are original blots, whereas they are small.

*In your cover letter, please note whether your blot/gel image data are in Supporting Information or posted at a public data repository, provide the repository URL if relevant, and provide specific details as to which raw blot/gel images, if any, are not available. Email us at plosone@plos.org if you have any questions.*

*4. Please include captions for your Supporting Information files at the end of your manuscript, and update any in-text citations to match accordingly. Please see our Supporting Information guidelines for more information:* [*http://journals.plos.org/plosone/s/supporting-information*](about:blank)*.*

*>>>>>*

The manuscript has been revised in accordance with the Supporting Information guidelines.

*[Note: HTML markup is below. Please do not edit.]*

*Reviewers' comments:*

*Reviewer's Responses to Questions*

***Comments to the Author*** *1. Is the manuscript technically sound, and do the data support the conclusions?

The manuscript must describe a technically sound piece of scientific research with data that supports the conclusions. Experiments must have been conducted rigorously, with appropriate controls, replication, and sample sizes. The conclusions must be drawn appropriately based on the data presented.*

*Reviewer #1: Yes*

*Reviewer #2: Partly*

*>>>>>>*

The manuscript has been revised accordingly.

*2. Has the statistical analysis been performed appropriately and rigorously?*

*Reviewer #1: Yes*

*Reviewer #2: No*

*>>>>>>>>*

The performance of the statistical analysis has been addressed in the revised manuscript according to the reviewer’s comment.

*3. Have the authors made all data underlying the findings in their manuscript fully available?

The* [*PLOS Data policy*](http://www.plosone.org/static/policies.action#sharing) *requires authors to make all data underlying the findings described in their manuscript fully available without restriction, with rare exception (please refer to the Data Availability Statement in the manuscript PDF file). The data should be provided as part of the manuscript or its supporting information, or deposited to a public repository. For example, in addition to summary statistics, the data points behind means, medians and variance measures should be available. If there are restrictions on publicly sharing data—e.g. participant privacy or use of data from a third party—those must be specified.*

*Reviewer #1: Yes*

*Reviewer #2: Yes*

*4. Is the manuscript presented in an intelligible fashion and written in standard English?

PLOS ONE does not copyedit accepted manuscripts, so the language in submitted articles must be clear, correct, and unambiguous. Any typographical or grammatical errors should be corrected at revision, so please note any specific errors here.*

*Reviewer #1: Yes*

*Reviewer #2: Yes*

*5. Review Comments to the Author

Please use the space provided to explain your answers to the questions above. You may also include additional comments for the author, including concerns about dual publication, research ethics, or publication ethics. (Please upload your review as an attachment if it exceeds 20,000 characters)*

*Reviewer #1: This is a nice work by Dr. Murakami and colleagues. The authors presented solid evidence demonstrating that TRIC-A, not Tric-b contributes to sinus atrial node pacemaking activity. Although the techniques used for all assays are routine, their data clearly support their conclusion that TRIC-A channel but not Tric-b is involved in cardiac rhythm formation and sympathetic nerve regulation of heart rate. Their data are of high quality. I have no any concerns or critiques.*

*>>>>>*

Thank you for your comments.

*Reviewer #2: General comments:
Murakami et al. provide evidence for a crucial role of TRICA in cardiac pace making. Using murine genetic knock-out models, the authors demonstrate significantly reduced heart rate as a result ofTRIC-A ablation. In vivo measurements as well as experiments in isolated cardiac tissue including electrophysiological recordings in cells from the SA region demonstrate that physiologic pace making as well as adrenergic control of cardiac functions is strictly dependent on TRICA expression. The study is carefully planned and the open questions about a role of TRICA in physiologic control over heart rate are addressed by adequate and complementary methods. However, I have several issues regarding data presentation and analysis. With respect to the conclusion, I feel that the still enigmatic mechanism underlying the cardiac function of TRICA needs more detailed discussion, and some of the results, especially the measurements of PKA-mediated phosphorylation deserve more attention.*

*>>>>>>*

We did not confirm a direct relationship between TRIC-A and PKA. Rather, we found decreased responsiveness to β-adrenergic stimulation in TRIC-A-deficient hearts. The manuscript has been revised according to the above comments.

*Major points:
1. The authors show convincingly that in TRIC-A -/- mice not only heart rate is lower and barely increases in response to beta-adrenergic stimulation, as compared to WT or TTIC-B +/-, but also beta adrenergic inotropism is largely eliminated. This is an intriguing finding and rather unexpected. The original hypothesis of the authors is that TRICA is part of the cardiac ER/SR Ca2+ handling machinery and as such part of the coupled clock in pacemaker cells. Although this is still plausible and may not be ignored, adrenergic signaling is obviously impaired at the level of PKA-dependent phosphorylation as demonstrated in Figure 1A for CREB. Since PKA-mediated phosphorylation is an essential mechanism of adrenergic control over the coupled clock machinery (see Vinogradova and Lakatta 2009; DOI: 10.1016/j.yjmcc.2009.06.014) the question arises: Can the authors exclude that the main role of TRICA is to enable proper PKA-dependent regulatory phosphorylation in cardiac cells? It is in any case very important to further strengthen the finding that genetic ablation of TRICA impairs PKA regulatory phosphorylation. Please include a more informative/ representative immunoblotting illustration together with a decent statistics on CREB phosphorylation. I strongly suggest to further corroborate this important finding also for other targets (e..g. phospholamban), and include a more in depth discussion as to how TRICA might interfere with PKA signaling. In this respect one need to consider that TRIC has recently been identified as an interaction partner of STIM1 to organize STIM/Orai complexes (Shrestha et al 2020/ DOI: 10.1371/journal.pbio.3000700), which in turn are coupled to local cAMP, PKA/Ca2+ signaling (Zhang et al 2019 /doi.org/10.1038/s41467-019-09593-0). Thus the authors may discuss the role of TRICA in SA cells beyond the original countercurrent concept or direct modulatory effects on RyR signaling.*

*>>>>>>>>>>>*

Intracellular calcium affects a number of channels, including HCN, CaV1.2, and NCX. The sinus node is complex and too small for detailed analysis. To determine the exact role of TRIC-A channels in PKA-dependent phosphorylation, it is necessary to perform analyses under simple conditions, such as TRIC-A overexpression in HEK293 cells. Nevertheless, this point is important.

We examined phosphorylation of phospholamban, which yielded results similar to those of the p-CREB analysis. Due to the low heart rate in TRIC-A^−/−^ mice, physiological analysis of the response to adrenergic stimuli may yield erroneous results. Physiological evaluation is usually dependent on the hypothesis that basal intracellular conditions are almost the same between wild-type control and TRIC-A^−/−^ mice. This may cause misinterpretation in TRIC-A^−/−^ mice.

Recently, Zhou et al., reported isoproterenol-induced arrhythmia in TRIC-A^−/−^ mice. They demonstrated a marginal effect of isoproterenol on heart rate, which was consistent with the results of the present study. In addition, they reported that chronic treatment with isoproterenol resulted in cardiac fibrosis in TRIC-A^−/−^ mice, which was reminiscent of chronic heart failure [Zhou et al., *Circulation Research.* 2020;126:417 – 435. Reference # 22 in the manuscript]. In their study, it seems likely that TRIC-A ablation resulted in exacerbated high sensitivity to β-adrenergic stimulation (arrhythmia and fibrosis), although basal heart rate was apparently lower than that of wild-type mice. If TRIC-A gene deletion causes high sympathetic responses under basal conditions, it may also cause a marginal response (i.e., increase in heart rate) to isoproterenol. Nevertheless, further studies are needed regarding the β-adrenergic cascade in TRIC-A^−/−^ mice.

*2. Along the same lines: STIM1 is a likely interaction partner of TRICA (Shrestha et al 2020). Moreover, STIM1/Orai signaling appears crucial (Zhang et al. 2015/ doi:10.1073/pnas.1503847112). Therefore, inspection of STIM1 and also Orai expression at the RNA level is recommended and the discussion should be extended towards a potential interference with the STIM/Orai pathway.*

>>>>>>>>>

We additionally examined expression of STIM1, Orai1, Orai2, and phospholamban (S1 Fig). We previously analyzed STIM1^+/−^ mice and found no significant changes in Heart Rate (HR) (Ohba et al., 2017, PLOS ONE | https://doi.org/10.1371/journal.pone.0187950).

*>>>>>>>>>>>
3. The authors repetitively claim that „sympathetic nerve regulation“ is modified/impaired. From my understanding, there is no solid evidence for a role of TRICA in sympathetic neurons? By contrast, the authors demonstrate in Figure 4 almost complete elimination of isoproterenol-induced positive inotropism in isloated atria. These impressive effects can hardly be explained by sympathetic nerve regulation. The authors need to explicitly discuss the evidence for a neuronal vs a muscular/cardiac mechanism and omit all misleading statements.*

>>>>>>>>>>>>>

TRIC-A^−/−^ mice have an obviously decreased heart rate, which is independent of blood pressure, as isolated hearts also showed a decreased heart rate. We have revised the text accordingly.

*Minor points:
1. The concentration of isproterenol (100 nM) should be stated also in the abstract.*

*>>>>>>>>>>>>>*

The manuscript has been revised according to the reviewer’s comment.

*>>>>>>>>>>>>>
2. Statistical analysis needs a more detailed description. What test(s) was/were used for comparison of multiple groups? The numbers of observations need to be stated consistently throughout the manuscript (eg. n is given with 9/10 in the text but stated as 6-15 in Figure 2A).*

*>>>>>>>>>>>>>*

The manuscript has been revised according to the reviewer’s comment.

*>>>>>>>>>>>>>
3. Specifically, statistics for immunoblotting data need extension. An N around 4 is in general insufficient for a decent statistical analysis. Statistical information, eg. as bar graphs, should be given in the respective figures.*

*>>>>>>>>>>>>>*

We performed additional analyses and presented bar graphs in S1 Fig of the revised manuscript.

*>>>>>>>>>>>>>
4. The designation of genetic models in text an figures is highly inconsistent – please use uniform terminology. Eg. In Figure 3, four(!) different terms are used for TRICA knock out mice: TricA-null, TRIC-A-null, TricA KO and TRIC-A- -/-.*

*>>>>>>>>>>>>>*

The manuscript has been revised according to the reviewer’s comment.

*>>>>>>>>>>>>>
5. In Figure 4, representative traces of contractile force are apparently of similar absolute amplitude and share the same scaling. However, the signal to noise ratio is substantially lower in the TRICA KO atria. Do the authors have any plausible explanation for this?*

*>>>>>>>>>>>>>*

Unfortunately, force contraction was unstable, although we tried to avoid vibration and other factors to prevent noise. Nevertheless, better contraction traces are presented in the revised manuscript.

*>>>>>>>>>>>>>
6. As above: Why are the TRIC-A -/- recordings in Figure 5 so noisy compared to WT?*

*>>>>>>>>>>>>>*

Accurate action potential recording is key to this study. The trace was replaced with a better one in the revised manuscript.

*>>>>>>>>>>>>>
7. The discussion may deserve remodeling – not only by including aspects mentioned above but also shortening other parts.*

*>>>>>>>>>>>>>*

The manuscript has been revised according to the reviewer’s comment.

*>>>>>>>>>>>>>
8. Supplementary data are not reasonably mentioned in the text. The authors should clearly indicate, mention the results illustrated in the supplement.*

*>>>>>>>>>>>>>*

The manuscript has been revised according to the reviewer’s comment.
